# Digital exercise interventions to improve physical functioning of people with breast cancer: Protocol for a scoping review

**DOI:** 10.1371/journal.pone.0350300

**Published:** 2026-06-01

**Authors:** Katherine A. Mankelow, Sean O’Connor, Olinda Santin, Nicole E. Blackburn, Iseult M. Wilson

**Affiliations:** 1 School of Nursing and Midwifery, Queen’s University Belfast, Belfast, United Kingdom; 2 School of Nursing and Paramedic Science, Ulster University, Belfast, United Kingdom; 3 School of Health Sciences, Ulster University, Derry~Londonderry, United Kingdom; La Trobe University - Bundoora Campus: La Trobe University, AUSTRALIA

## Abstract

**Introduction:**

Breast cancer incidence rates are rising due to early detection, improved screening methods and advances in treatment options. As mortality rates reduce, there is a growing population living longer with treatment side-effects who require assistance. Prevalent issues arising after breast cancer treatment include pain, fatigue, lymphoedema and arm and shoulder mobility issues. Evidence demonstrates that upper limb exercises help reduce these common side effects and improve day-to-day functioning and quality of life. However, access to physiotherapy and rehabilitation services remains limited due to resource and access constraints. Despite a growing interest in digital health resources, little is known about the effectiveness of using digital exercise interventions to help resolve this problem.

**Methods and analysis:**

A scoping review of available literature will be undertaken to explore what digital or online prehabilitation or rehabilitation interventions exist for people with breast cancer that incorporate an exercise component aimed at improving physical function or mobility. Peer-reviewed studies in English will be eligible for inclusion. A systematic search of Medline ALL, Embase, CINAHL, and Web of Science will be conducted and any relevant grey literature form trusted sources will also be included in our review. The Arksey and O’Malley framework will be applied together with updated guidance from other authors. To enhance rigour, the Joanna Briggs Institute methodology for scoping reviews will also be used to ensure accurate reporting. Articles will be screened by title and abstract against eligibility criteria before independent full text screening by two researchers. Arising conflicts will be resolved by consulting a third reviewer. To summarise the available evidence, data will be extracted using a tailored charting template and a descriptive narrative synthesis will follow.

**Ethics and dissemination:**

As this research involves the analysis of already published, peer-reviewed literature, ethical approval is not required. The results of this scoping review will be submitted for publication in a peer-reviewed journal. Any significant deviations from the original protocol will be transparently reported and justified.

## Introduction

Breast cancer has the highest global age-standardised incidence rate and was the second most commonly diagnosed cancer in 2020, with 2.3 million new cases reported [1]. As a result, there is a growing demand on the health service to provide breast cancer patients with lifesaving treatment. Surgery, radiotherapy and systematic anti-cancer treatments are commonly used to treat breast cancer, and treatment choice will depend on the type of breast cancer, tumour location, patient preference, and how advanced the disease is [2]. It is not uncommon for patients to experience negative side-effects associated with their cancer treatments, and with many patients now living longer after diagnosis, it is important to help support them with these challenges [3,4].

Often a form of surgery will be recommended by clinicians as a first line treatment. People undergoing surgery for breast cancer can experience pain, lymphoedema, and arm and shoulder mobility issues that can all affect their normal functioning, work, and quality of life [5]. Upper limb rehabilitation has been shown to help reduce arm and shoulder morbidity following surgery through stretches, and range of motion and strengthening exercises [5–7]. Unfortunately, due to a lack of high quality RCT studies on this topic, there is currently no unanimous consensus on how best to deliver these exercises. It is therefore unsurprising that the recently updated NICE guidelines for the treatment of breast cancer show a preference for individualised in-person rehabilitation programs [2]. However, this may not always be feasible with available resources and current pressures facing healthcare systems around the world.

It is estimated that less than a third of patients have access to rehabilitation support following breast cancer surgery [8]. With more people being diagnosed and cancer care waiting times generally increasing [9], timely access to postoperative physiotherapy and exercise support is often limited, which can delay recovery and contribute to unmet rehabilitation needs [4,5,8]. Digital health interventions have emerged as a promising way to provide remote, structured and scalable exercise-based rehabilitation [10,11]. However, there is currently no synthesis of digital exercise-based rehabilitation resources that specifically report outcomes related to physical functioning of the arm and shoulder after breast cancer treatment. Therefore, this scoping review will assess the existing literature to identify such resources and help inform the development and implementation of future digital interventions, with the aim of improving access to rehabilitation and supporting better daily functioning and quality of life.

## Aim

The main objective of this scoping review is to examine what is known about the effectiveness of existing digital exercise rehabilitation resources on physical functioning in individuals with breast cancer. The review seeks to identify, describe, and appraise these resources to map the current evidence base and highlight gaps that may inform future research and clinical practice.

## Review question

This scoping review seeks to address the following research question:

What types of digital interventions incorporating exercise exist to support prehabilitation and/ or rehabilitation among breast cancer patients?

## Materials and methods

### Design

A scoping review design has been selected to capture the broad and emerging evidence in this field. This approach allows for a comprehensive mapping of digital exercise interventions, providing an overview of their key characteristics and identifying gaps in the literature.

### PCC framework

The Population, Concept and Context framework recommended in the JBI Manual for Evidence Synthesis [12] was used to identify the following key concepts:

- Population: People with breast cancer
- Concept: Pre- and/ or rehabilitation programs with a focus on exercise
- Context: Digital intervention

## Methods

The Arksey and O’Malley framework will be used to help plan and conduct the search together with updated guidance from other authors [13–15]. This method was chosen because its structured stages, including stakeholder consultation, support the integration of public and patient involvement, enhancing the relevance and applicability of findings.

The Joanna Briggs Institute methodology for scoping reviews [12] and the Preferred Reporting Items for Systematic Reviews and Meta Analysis for Scoping Reviews (PRISMA-ScR) checklist [15] will be applied to accurately report our findings. The completed PRISMA‑P checklist is provided in S1 Appendix.

As the evidence base for digital exercise‑based interventions in breast cancer is still emerging, with relatively few RCTs to date, a scoping review approach is appropriate. For this reason, a broad range of study designs will be included to capture the full scope of existing evidence.

### Eligibility criteria

Results of a rapid review by the authors identified limited evidence on surgical interventions alone. Therefore, the search was broadened to include all treatment interventions incorporating an exercise component, to better reflect the complexity of cancer rehabilitation and the emerging range of digital interventions. Given that patients often experience both physical and psychological challenges affecting their wellbeing and quality of life [16], many interventions adopt a multimodal approach. Based on this, the following eligibility criteria have been defined.

Studies will be included if they meet the following criteria:

Inclusion criteria:

- Adults with a diagnosis of breast cancer at any stage of the treatment or survivorship pathway
- Studies involving mixed cancer populations will be included only if breast cancer–specific data can be extracted separately
- Prehabilitation or rehabilitation resources
- Interventions aimed at improving physical functioning and mobility
- Must include exercise component
- Interventions must be digital or online (home-based), including (but not limited to) mobile applications, web-based platforms, online videos, virtual or telehealth programmes, or digital interventions supported by wearables
- Interventions must be delivered digitally to support independent or self-managed rehabilitation
- RCT’s, cohort studies, mixed-methods, qualitative studies, feasibility studies, and quasi-experimental studies
- Protocols that are relevant but do not yet have published results
- Peer-reviewed articles in English
- No restrictions on publication date

Studies will be excluded if they meet the following criteria:

Exclusion criteria:

- Studies including other types of cancer without separate data on breast cancer
- Studies that do not include an exercise component
- Studies focused exclusively on palliative care
- Studies focused on breast cancer prevention or preventing reoccurrence of breast cancer
  - Weight loss or weight management resources
  - Walking only interventions
- Fully supervised, in-person programmes (non-digital interventions)
- Scoping and systematic reviews
- Opinion pieces, editorials, and conference abstracts

### Search strategy

The search strategy was developed in consultation with the research team and subject librarian, and guided by the PCC framework, focusing on three key concepts: breast cancer, exercise, and digital approaches. Trial searches were conducted in each database to refine the use of subject headings, keywords, Boolean operators, and wildcards, ensuring a comprehensive search.

Tables 1–4 present our planned search strategy for each individual database. The subject headings and keywords within each concept will be combined using OR, and the overall concepts with AND, to capture articles addressing all three areas.

**Table 1:** Search strategy for Medline ALL (Ovid).

Concept	Subject Headings (MeSH)	Keywords
Breast Cancer	Breast Neoplasms/ Breast Cancer Lymphedema/ Carcinoma, Ductal, Breast/	breast cancer*.mp. breast tumo?r*.mp. breast carcinoma*.mp.
Exercise	Exercise/ Exercise Therapy/ Physical Therapy Modalities/ Resistance Training/ Preoperative Exercise/	exercise therap*.mp. resistance training.mp. muscle stretch*.mp. muscle strength*.mp. prehab*.mp. preoperative exercis*.mp. postoperative exercise.mp. rehab*.mp.
Digital	Digital Health/ Telemedicine/ Telerehabilitation/ Internet-Based Intervention/	online.mp. digital.mp. home-based.mp. website.mp. virtual.mp. mhealth.mp. digital health.mp. telemedicine.mp. telerehabilitation.mp. telehealth.mp. internet-based intervention.mp.

**Table 2:** Search strategy for Embase (Ovid).

Concept	Subject Headings (Emtree)	Keywords
Breast Cancer	breast tumor/ breast cancer-related lymphedema/ breast ductal carcinoma/ breast cancer/ breast carcinoma/	breast cancer*.mp. breast tumo?r*.mp. breast carcinoma*.mp.
Exercise	exercise/ kinesiotherapy/ physiotherapy/ resistance training/ preoperative exercise/ muscle stretching/ muscle strength/ preoperative exercise/ rehabilitation/	exercise therap*.mp resistance training.mp muscle stretch*.mp. muscle strength*.mp. prehab*.mp. preoperative exercis*.mp. postoperative exercis*.mp rehab*.mp
Digital	digital health/ telemedicine/ telerehabilitation/ telehealth/ web-based intervention/	online.mp. digital.mp. home-based.mp. website.mp. virtual.mp. mhealth.mp. digital health.mp. telemedicine.mp. telerehabilitation.mp. telehealth.mp. internet-based intervention.mp.

**Table 3:** Search strategy for CINAHL (EBSCOhost).

Concept	CINAHL Subject Headings	Keywords
Breast Cancer	(MH “Breast neoplasms”) (MH “Carcinoma, ductal, breast”)	“breast cancer*” “breast tumo#r*” “breast carcinoma*” “breast cancer lymphed#ema”
Exercise	(MH “Exercise”) (MH “Therapeutic Exercise”) (MH “Physical Therapy”) (MH “Resistance Training”) (MH “Prehabilitation”)	“exercise therap*” “resistance training” “muscle stretch*” “muscle strength*” “prehab*” “preoperative exercis*” “postoperative exercis*” “rehab*”
Digital	(MH “Digital Health”) (MH “Telemedicine”) (MH “Telerehabilitation”) (MH “Telehealth”) (MH “Internet-Based Intervention”) (MH “World Wide Web Applications”)	“online” “digital” “home-based” “website” “virtual” “digital health” “telemedicine” “telerehabilitation” “telehealth” “internet-based intervention”

**Table 4:** Search strategy for Web of Science (Clarivate).

Concept	Subject Headings	Keywords
Breast Cancer	N/A	“breast cancer*” “breast tumo$r*” “breast carcinoma*” “breast neoplasms” “breast cancer lymphed$ema” “ductal breast carcinoma”
Exercise	N/A	exercise “physical therap*” “resistance training” “muscle stretch*” “muscle strength*” prehab* “preoperative exercis*” “postoperative exercis*” rehab*
Digital	N/A	online digital home-based website virtual telemedicine telerehabilitation telehealth “internet-based intervention”

### Information Sources

The following databases will be searched to uncover relevant literature: Medline ALL, Embase, CINAHL, and Web of Science. Trial registration sites (ClinicalTrials.gov, ISRCTN registry and EU Clinical Trials Register) will also be explored to ensure we include any relevant upcoming trials that meet eligibility criteria. In addition, citation searches will further help identify any relevant grey literature that may be missed. Reference lists from excluded reviews on the same topic will be individually searched to identify additional primary sources. Any additional findings will subsequently be added to the screening process of this review.

### Data collection, extraction and analysis

An initial screening of titles and abstracts will be conducted by one reviewer to remove clearly irrelevant studies. All potentially eligible records will then be independently screened at the full-text level by two reviewers. Disagreements will be resolved through discussion or by consulting a third reviewer until consensus is reached.

All search results will be imported to Covidence [17] for management and tracking of the screening process. The study selection process will be documented using a PRISMA flow diagram [15]. Included studies will be exported to EndNote (version 21.5, Clarivate Analytics [18] to support citation management and facilitate reference checking.

To accurately synthesise the available evidence, data will be extracted using a tailored charting template from Covidence and summarised in a spreadsheet. To enhance accuracy and rigour, the modified extraction template will be reviewed by the research team and refined as needed to ensure all relevant data are captured prior to full data extraction. This iterative process will help ensure clarity and consistency throughout.

Extracted data will include: general study information (e.g., author, publication year, country), study aims, design, duration, funding source, and any reported conflicts of interest. Participant information will include eligibility criteria, recruitment method, sample size, age, gender, ethnicity, breast cancer type, and cancer treatments received.

Intervention‑related data will include the delivery mode, exercise components, prescribed intensity, frequency and duration, additional treatments, setting, required equipment, and any multimodal components. We will also extract information on intervention development processes, such as whether co‑design or stakeholder involvement was used, or note where these details are absent. With respect to outcomes, we will extract primary and secondary outcomes as defined by each study; the physical functioning domains assessed (e.g., range of motion, strength, mobility, daily functioning); the measurement tools used; whether these tools are validated; and, where available, whether they have been validated specifically in breast cancer populations. Measurement timepoints and any reported usability or acceptability data will also be recorded. Missing or unspecified data will be documented.

In this scoping review, appraisal refers to the comprehensive mapping and characterisation of intervention features as outlined above, rather than an assessment of methodological quality or risk of bias. Consistent with scoping review methodology, no formal critical appraisal of study quality will be conducted. To help summarise all of the findings a narrative synthesis of the collected data and results will follow. Patterns and themes will be identified to summarise the range and scope of existing digital exercise rehabilitation interventions for breast cancer patients. Where applicable, gaps in the literature and areas for future research will be highlighted.

## Supporting information

S1 Appendix PRISMA‑P Checklist. Description: Completed PRISMA‑P checklist for protocol reporting.
(DOCX)
