# Supplementary material for: Digital exercise interventions to improve physical functioning of people with breast cancer: Protocol for a scoping review
Source: PLoS One. 2026 Jun 1;21(6):e0350300. doi: 10.1371/journal.pone.0350300 (PMC13225433; doi:10.1371/journal.pone.0350300)
Supplement: S1 Appendix — Description: Completed PRISMA‑P checklist for protocol reporting. (DOCX) [file pone.0350300.s001.docx]

**PRISMA-P (Preferred Reporting Items for Systematic review and Meta-Analysis Protocols) 2015 checklist: recommended items to address in a systematic review protocol***

| Section and topic | Item No | Checklist item |
| --- | --- | --- |
| ADMINISTRATIVE INFORMATION | | |
| Title: |  |  |
| Identification | 1a | Identify the report as a protocol of a systematic review *– This manuscript is a protocol for a scoping review (see title)* |
| Update | 1b | If the protocol is for an update of a previous systematic review, identify as such *– N/A as this is a new review* |
| Registration | 2 | If registered, provide the name of the registry (such as PROSPERO) and registration number *– This scoping review protocol is registered on the Open Science Framework (OSF):* [*https://osf.io/n7pbd/?view_only=0640b0bb2abd4d6893901089bcb67283*](https://protect.checkpoint.com/v2/r02/___https://osf.io/n7pbd/?view_only=0640b0bb2abd4d6893901089bcb67283___.YzJlOnVsc3RlcnVuaXZlcnNpdHk6YzpvOmM5NzY1MWFhZWRhYzc5NjE3ZWE0OGI1ZDFmODI0ZmI2Ojc6NWM3NTo4YzRlNzk1MGIxN2NiMmMxZTk5M2JmZDBkM2NiNWQ3MjQxYjIwMzQ3ODVhMGY0MmI4YjE4MWM1MmJlZGFkZjJlOnA6VDpO)*.* |
| Authors: |  |  |
| Contact | 3a | Provide name, institutional affiliation, e-mail address of all protocol authors; provide physical mailing address of corresponding author *– Names, affiliations and correspondence email address are on the title page of the protocol. Physical mailing address omitted in accordance with current digital communication practices.* |
| Contributions | 3b | Describe contributions of protocol authors and identify the guarantor of the review – *All authors contributed to the development of the protocol. Katherine A. Mankelow is the guarantor.* |
| Amendments | 4 | If the protocol represents an amendment of a previously completed or published protocol, identify as such and list changes; otherwise, state plan for documenting important protocol amendments *– N/A as this is the first version. Any amendments will be documented in the OSF record.* |
| Support: |  |  |
| Sources | 5a | Indicate sources of financial or other support for the review *– Department for the Economy (DfE) Co-operative Awards in Science and Technology (CAST) studentship and Cancer Focus NI, reported on page 15 under Funding and Sponsorship* |
| Sponsor | 5b | Provide name for the review funder and/or sponsor – *See page 15* |
| Role of sponsor or funder | 5c | Describe roles of funder(s), sponsor(s), and/or institution(s), if any, in developing the protocol *– See page 15* |
| INTRODUCTION | | |
| Rationale | 6 | Describe the rationale for the review in the context of what is already known – *See Introduction section on page 3-5* |
| Objectives | 7 | Provide an explicit statement of the question(s) the review will address with reference to participants, interventions, comparators, and outcomes (PICO) *– See Review question on page 5 which follows the PCC framework as this is better suited to the aims of this scoping review. The review will address the following question: “What types of digital interventions incorporating exercise exist to support prehabilitation and/ or rehabilitation among breast cancer patients?”* |
| METHODS | | |
| Eligibility criteria | 8 | Specify the study characteristics (such as PICO, study design, setting, time frame) and report characteristics (such as years considered, language, publication status) to be used as criteria for eligibility for the review – *See Eligibility criteria on page 7-8 for detailed information on the in- and exclusion criteria* |
| Information sources | 9 | Describe all intended information sources (such as electronic databases, contact with study authors, trial registers or other grey literature sources) with planned dates of coverage *– See Information Sources on page 13 for details on selected electronic databases and grey literature management* |
| Search strategy | 10 | Present draft of search strategy to be used for at least one electronic database, including planned limits, such that it could be repeated – *See the Search Strategy section on pages 8–12, which includes four tables outlining tailored search strategies for all proposed electronical databases. These strategies include subject headings, keywords, Boolean operators, and planned limits to ensure reproducibility.* |
| Study records: |  |  |
| Data management | 11a | Describe the mechanism(s) that will be used to manage records and data throughout the review – S*ee Data collection, extraction and analysis on pages 13-14, which describes how records will be managed using Covidence and Endnote* |
| Selection process | 11b | State the process that will be used for selecting studies (such as two independent reviewers) through each phase of the review (that is, screening, eligibility and inclusion in meta-analysis) *– See Data collection, extraction and analysis on pages 13-14 for full details on the screening process and selection strategy, which has been developed to minimise bias.*  *In summary, one initial reviewer will conduct initial title and abstract screening, after which two independent reviewers will screen full texts, resolving disagreements through third-party consultation and discussion until consensus is reached.* |
| Data collection process | 11c | Describe planned method of extracting data from reports (such as piloting forms, done independently, in duplicate), any processes for obtaining and confirming data from investigators *– See Data Collection, Extraction and Analysis on pages 13–14, which outlines the planned process. A modified charting template will be used to systematically extract relevant data from included studies. An iterative approach will be used, allowing the template to be refined as needed throughout the review. Data will be extracted independently by two reviewers, and any discrepancies will be resolved through discussion or by consulting a third reviewer.* |
| Data items | 12 | List and define all variables for which data will be sought (such as PICO items, funding sources), any pre-planned data assumptions and simplifications *– Data extraction will focus on general study information and study characteristics, including methods, participants, type of intervention and outcomes. See Data Collection, Extraction and Analysis on pages 13–14 for full details.* |
| Outcomes and prioritization | 13 | List and define all outcomes for which data will be sought, including prioritization of main and additional outcomes, with rationale. *– As this is a scoping review, we aim to map outcomes across studies rather than evaluate them quantitatively. Outcomes of interest are focused on their effect on physical functioning (positive, neutral or negative).* |
| Risk of bias in individual studies | 14 | Describe anticipated methods for assessing risk of bias of individual studies, including whether this will be done at the outcome or study level, or both; state how this information will be used in data synthesis *– N/A* |
| Data synthesis | 15a | Describe criteria under which study data will be quantitatively synthesised – *N/A* |
|  | 15b | If data are appropriate for quantitative synthesis, describe planned summary measures, methods of handling data and methods of combining data from studies, including any planned exploration of consistency (such as I^2^, Kendall’s τ) *– N/A* |
|  | 15c | Describe any proposed additional analyses (such as sensitivity or subgroup analyses, meta-regression) – *N/A* |
|  | 15d | If quantitative synthesis is not appropriate, describe the type of summary planned *– Data will be synthesised descriptively using tables and narrative summary. No quantitative synthesis is planned.* |
| Meta-bias(es) | 16 | Specify any planned assessment of meta-bias(es) (such as publication bias across studies, selective reporting within studies) – *N/A* |
| Confidence in cumulative evidence | 17 | Describe how the strength of the body of evidence will be assessed (such as GRADE) – *N/A* |

*** It is strongly recommended that this checklist be read in conjunction with the PRISMA-P Explanation and Elaboration (cite when available) for important clarification on the items. Amendments to a review protocol should be tracked and dated. The copyright for PRISMA-P (including checklist) is held by the PRISMA-P Group and is distributed under a Creative Commons Attribution Licence 4.0.**

*From: Shamseer L, Moher D, Clarke M, Ghersi D, Liberati A, Petticrew M, Shekelle P, Stewart L, PRISMA-P Group. Preferred reporting items for systematic review and meta-analysis protocols (PRISMA-P) 2015: elaboration and explanation. BMJ. 2015 Jan 2;349(jan02 1):g7647.*
